# Rare ophthalmology diseases

**Published:** 2019

**Authors:** Elena Angelica Sburlan, Liliana-Mary Voinea, Cristina Alexandrescu, Sanziana Istrate, Raluca Iancu, Ruxandra Pirvulescu, Aida Geamanu, Mihai Ghita, Emil Ungureanu, Ciuluvica Radu

**Affiliations:** * Ophthalmology Department, Bucharest University Emergency Hospital, Bucharest, Romania; ** Anatomy Department, “Carol Davila” University of Medicine and Pharmacy, Bucharest, Romania

**Keywords:** rare, ocular, pathology, registries, treatment, FDA, systemic, disease, orphan

## Abstract

Rare ocular pathology has an important impact on the quality of life of patients because often the damage is bilateral and, although asymmetric, causes a significant decrease in visual acuity. Because it may be asymptomatic until a relatively late stage, diagnosis is frequently delayed. A general understanding of the disease pathophysiology, diagnosis, and treatment may assist primary care physicians in referring high-risk patients for comprehensive ophthalmological examination and for a more active involvement in their care. Moreover, a significant percentage of these orphan diseases do not have treatment approved by the FDA.

The examination and monitoring of patients with rare ophthalmological disorders represents a key component of an ongoing project at the University Emergency Hospital, Bucharest, Romania – Ophthalmology Clinic. Rare disease registries are leading tools for the development of clinical research for rare diseases, improvement of patient access to new diagnostic methods, follow-up and new emerging therapies.

As of this moment, the European list of rare diseases includes 53 ophthalmological diseases, which are classified as rare diseases and another 103 systemic diseases with ophthalmological involvement, out of a total of 7000 rare diseases.

## Introduction

A patient experiencing a permanent loss of sight requires medical support, psychological rehabilitation, and social reintegration in order to gain a normal quality of life. Patients with rare diseases often have similar problems, such as lack of qualitative information, delayed diagnosis, as well as a lack of access to treatment and care. The main concern for rare diseases is relatively new in Romania and most patients presenting with these conditions are misdiagnosed, poorly treated and require multiple secondary opinions and treatments. For these reasons, the health care system requires specialists in these diseases as well as adequate resources to further investigate this field. The vast majority of rare diseases have a genetic origin, thus being present throughout the individual’s life even if the symptoms are not present since childhood. As such, screening, diagnosis, and patient identification are the key elements for the optimal management strategy in these pathologies. 

Primary care physicians play an important role in the diagnosis of rare ophthalmological diseases by referring patients with positive family history or with suspicious findings for complete ophthalmologic examination. They can improve treatment outcomes by reinforcing the importance of medication adherence. Early identification and immediate management of eye related pathologies should commence as soon as possible, while diagnosis and prompt intervention have a significant impact on the prognosis for many potentially blinding disorders. If left undetected and untreated, such problems may potentially lead to irreversible vision damage with subsequent lack of self-confidence and difficulties in educational achievement and job opportunities.

## Registries of rare diseases

The registries of rare diseases represent an important approach for gathering epidemiological information and relevant samples for clinical research while being essential for feasibility studies, especially for enrollment in clinical trials and the establishment of treatment protocols.

Once these databases of rare diseases are created [**1**], constant optimization and modification can be made by all invested parts (researchers, pharmaceutical industry, biotech companies, physicians but also patients and patients associations) both for research and for clinical practice. The European collection of data for diseases includes 651 registries of which 454 are national registries, 77 are regional registries, 45 are European registries, 71 are global registries, and 4 are undefined [**2**]. France has a total of 132 rare disease registries (5 of which are ophthalmological disease registries), Germany has a total of 116 registries (2 of which are ophthalmological disease registries and 5 of which are systemic disease with ocular involvement registries), Spain has a total of 46 rare diseases registries (2 of which are ophthalmological rare disease registries), Bulgaria has 11 rare disease registries and Romania currently has only two national rare disease registries, none of which is dedicated to eye diseases. Out of 45 European registries, 5 are related to rare ocular diseases but none of them address the corneal pathology. 

Out of all the patients admitted to the ophthalmology ward of the Bucharest University Emergency Hospital between 2009-2016, 3.6% had rare corneal dystrophies, 10% of the hospitalized uveitis was part of juvenile idiopathic arthritis (rare immunologic disease), and 2% were retinal dystrophies associated with genetic transmission. Our clinic presents increased addressability at a national level for rare ocular diseases.

## Classification of Rare Diseases

Although more than 7,000 rare diseases have been identified, medical professionals do not often recognize or encounter orphan disease patients [**1**].

The World Health Organization – ICD classification (International Classification of Diseases) contains almost 500 rare diseases, is based on morbidity [**5**] and it is mainly used in Europe [**3**].

The International Health Terminology Standards Development Organization – SNOMED CT classification (The Systematizes Nomenclature of Medicine Clinical Terms) used in England and in more than 50 others countries includes nearly 3000 orphan diseases with specific encodings.

Orphanet is the most comprehensive online database of rare diseases. It contains about 7000 of such pathologies with specific encodings (Orpha code). It provides information on the symptoms of orphan diseases, identified genetic mutations and currently used orphan drugs [**4**].

A compelling number of rare diseases can lead to eye disorders and as some of these are extremely uncommon, inclusion of each eye related condition is beyond the scope of this article. Consequently, in this review article, we have chosen to target only several ophthalmological rare disorders and their affiliate findings.

## Achromatopsia

Achromatopsia is a rare, autosomal recessive ocular disease characterized by the inability to perceive colors, nystagmus, photophobia, and severely reduced visual acuity due to the absence or impairment of cone cell function [**7**]. 

Worldwide, achromatopsia prevalence is estimated at approximately 1/ 30.000 – 1/ 50.000 newborns [**8**]. The disease is detected at around 6 months of age through severe photophobia and pendular nystagmus. At 7-8 years old, nystagmus becomes less noticeable and other symptoms of the disease become more relevant [**8**]. Usually, affected individuals have very low visual acuity and a reduced or complete lack of ability to distinguish colors associated with hemeralopia. In addition, a small central scotoma can be detected. Fundus examination is within normal limits. Most individuals exhibit total achromatopsia with the absence of function for all three types of cones. Incomplete achromatopsia cases present with similar but less severe symptoms [**6**].

The diagnosis of achromatopsia is raised on ophthalmic examination, color vision testing and electrophysiological tests (electroretinogram –ERG) which show the absence of photopic response [**9**] and a normal scotopic response. Optical coherence tomography detects progressive disruption and/or loss of photoreceptor function in the internal and external retinal layer associated with a diminution in retinal pigment epithelium [**10**]. Diagnosis is confirmed by molecular genetic testing. Five genes [GNAT2 (1p13), PDE6C (10q24), PDE6H (12p13), CNGA3 (2q11.2), and CNGB3 (8q21.3)] were associated with achromatopsia, all of them encoding key components of the photo transduction cascade of cones [**11**]. Currently, there is no specific therapy and the management of the disease is symptomatic. Patients should be informed about the use of glasses with filters to reduce photophobia and improve contrast sensitivity [**12**]. There are devices with great magnifiers for reading. Since 2003 there has been a cybernetic device called eyeborg that allows people to perceive color by sound waves. 

Achromatopsia is an autosomal recessive disease [**13**]. Once the responsible gene has been identified, testing for the family members is required. Genetic counseling with proper information should be offered to couples at risk (if both individuals are carrying a mutation-causing gene there is a 25% chance of passing it to their offspring) [**14**].

## Cone-Rode dystrophies (CRD)

Rod and cone cell dystrophies are included in the pigmentary retinopathies with genetic transmission [**15**]. During the final stages of the disease, several retinal dystrophies (choroideremia, Stargardt macular dystrophy, Sorsby dystrophy) usually involve the entire retinal surface and may be erroneously diagnosed as pigmentary retinopathies [**16**].

Autosomal dominant, recessive, and X-inked transmissions have been described. Recent molecular genetic studies identified 16 different genomic regions, each one containing a gene implicated in cone and rode dystrophies. CRD prevalence is estimated to be 1:40.000. There is insufficient data in the literature about this dystrophy, although it has been suggested that this affection could be more common than originally thought [**17**]. Moreover, some patients, which are diagnosed with pigmentary retinopathy, may associate more involvement of the cone cells and this leads to a challenging differential diagnosis between Cone-Rode dystrophies and retinitis pigmentosa. A recent study that included 278 patients with pigmentary retinopathy with recorded electroretinograms and well-measured rod levels showed that 41% had a bigger deficit of cone relative to rod cells.

CRD is characterized by early vision loss and color blindness associated with a progressive deficit of the visual field. Patients usually present a more severe loss of cone than rod cells. In these cases, initial symptomatology is characterized by loss of central vision acuity, loss of color perception and photophobia. When rod cell involvement progresses, affected people experience a decrease in night vision and a decrease in peripheral vision. In rare cases, simultaneous damage to rod and cones occurs and the symptoms begin around the same time. Diagnosis of CRD is based on clinical history, fundus examination, and electroretinogram [**18**]. For some genes, there is the possibility of genetic molecular diagnosis. Even from the onset phase, a diagnosis can be made before peripheral field deficits or retinal peripheral abnormalities become apparent. Currently, there is no therapy to stop the progression of the disease and the prognosis is unfavorable. Management aims at slowing the degenerative process, treating complications, and helping patients adapt to the social and psychological impact of blindness.

## Bonnet–Dechaume–Blanc

(Wyburn Mason syndrome/ racemosa angioma) is a rare, congenital, arteriovenous, nonhereditary, malformation of the eye and brain, typically involving the optic disc or retina and the midbrain [**19**]. Ocular manifestations are congenital, usually unilateral but may start to appear only during childhood. The typical lesion consists of markedly dilated and tortuous vessels that shunt blood flow directly from arteries to veins. There are three categories of arteriovenous malformations that are categorized depending on the severity of the malformation. The first category consists of the patient having small lesions that are usually asymptomatic. The second category, more severe, is represented by a malformation, which is missing a connecting capillary. The capillary is the link between an artery and a vein and when it is absent, edemas, hemorrhages, and visual impairments can occur. The third and most severe category represents a severe malformation in which there is a great degree of dilated vessels such that there is almost no macroscopic distinction between arteries and veins. When the symptoms are this severe, the patient has a significantly increased risk of developing vision loss [**20**]. Visual acuity ranges from normal to markedly reduced in the involved eye. Intraocular haemorrhage and secondary neovascular glaucoma are possible complications. No treatment is indicated for primary lesions. Associated skin lesions are present in a small number of cases, usually on the face. These lesions can vary from slight discoloration to extensive nevi and angiomas. There are fewer than 100 reported cases of patients with Wyburn Mason Syndrome.

## Conclusions

- Eyesight is contemplated by many as the most significant of the basic senses. Loss of vision can have huge repercussions on the quality of life. 

- 1 in 10 people are affected by rare diseases, 

- 1 of 2 patients diagnosed with a rare disease is a child, 

- 350 million people suffer from a rare disease globally, 

- 8 in 10 rare diseases are caused by a faulty gene, 

- 8 years is the average time it takes for rare patients to receive an accurate diagnosis, 

- 95% of the rare diseases lack an FDA Approved Treatment [**21**].

**Acknowledgements**

The authors would like to thank the Ophthalmology Department and the medical staff from “Carol Davila” University of Medicine and Pharmacy, Bucharest, Romania and from the University Emergency Hospital, Bucharest, Romania. All authors have equally contributed for the development and publication of this review.

**Conflict of interests**

The authors declare no conflict of interest.
